# Quantitative comparison between amyloid deposition detected by 99mTc-diphosphonate imaging and myocardial deformation evaluated by strain echocardiography in transthyretin related cardiac amyloidosis

**DOI:** 10.1186/1750-1172-10-S1-P47

**Published:** 2015-11-02

**Authors:** Gianluca Di Bella, Fabio Minutoli, Anna Mazzeo, Claudia Stancanelli, Luca Gentile, Sergio Baldari, Scipione Carerj, Giuseppe Vita

**Affiliations:** 1 University of Messina, Clinical and experimental medicine department, 98100, Messina, Italy; 2 University of Messina, Department of Biomedical Sciences and of Morphologic and Functional Images, 98100, Messina, Italy; 3 University of Messina, Department of Neurosciences, 98100, Messina, Italy

## Aims

The aim of our study is to assess the effect of amyloid deposition on myocardial function.

## Methods and results

28 patients with transthyretin mutation and a group of 14 controls underwent echocardiography to quantify left ventricular (LV) dimensions and function, and global (G) longitudinal (L), radial (R) and circumferential (C) strain (S). 99mTc-3, 3-diphosphono-1, 2-propanodicarboxylic-acid-scintigraphy (99mTc-DPD) was used to quantify cardiac amyloidosis (CA). 99mTc-DPD revealed accumulation in 14 of 28 patients (CA-group) and no accumulation (no CA-group) in 14 patients. Cardiac accumulation was mild-moderate in 5 (Mild-Moderate CA-group) and severe in 9 patients (Severe CA-group). Severe CA-group showed higher values of LV septal thickness (LVST), posterior wall thickness and E/E’ ratio than the no CA-group and the control group (adj. p<0.05). Ejection fraction was similar among groups (p=0.65). GLS was lower (p=<0.001) in severe CA-group (-12.2 ± 4.5) respect to no CA-group (-19.3 ± 3.0) and to the control group (-20.9 ± 2.5). On the contrary, GCS and GRS were lower (p=<0.05) in mild-moderate CA-group (-10.8 ± 4.1 and 9.5 ± 5.7, respectively) respect to the severe CA-group (-18.9 ± 5.1 and 23.9 ± 6.3 respectively), no CA-group (-19.2 ± 4.1 and 28.4 ± 10.2 respectively) and the control group (-23.9 ± 4.4 and 29.9 ± 8.7 respectively). A correlation was found between the scintigraphic heart retention (HR) index and LVST (ρ=0.72; p<0.001) and E/E’ (ρ=0.46; p=0.03). An inverse tendency was observed between HR and GLS (ρ=−0.40; p=0.06).

## Conclusions

99mTc-DPD HR is well correlated with LVST, diastolic and longitudinal systolic dysfunction.

